# The Efficacy of Intermittent Walking in Water on the Rate of MHPG Sulfate and the Severity of Depression

**Published:** 2011

**Authors:** Valiollah Dabidy Roshan, Mehdi Pourasghar, Zeinab Mohammadian

**Affiliations:** 1 College of Physical Education and Sport Sciences, Department of sport Physiology, University of Mazandaran, Babolsar, Iran.; 2 Psychiatry and Behavioral Sciences Research Center, Mazandaran University of Medical Sciences, Sari, Iran.

**Keywords:** Aerobics, Depression, Hamilton scale, MHPG Sulfate

## Abstract

**Objective:** Many studies evaluated the efficacy of exercise on some depression indices, but the effect of physical exercise in exhilarating milieu on urine 3-Methoxy-4-Hydroxyphenylglycol** (**MHPG) sulfate-the main metabolite of norepinephrine is not clear. The purpose of this research was to study the effect of a six-week intermittent walking in the water on 24-hour urine MHPG sulfate in depressed female teenagers.

**Methods:** Twenty-four high school female students with depression were divided randomly into case and control group. Pool walking exercise program was implemented 3 sessions weekly for 6 weeks and with a rate of 60-70% of the maximum heart rate. The control group didn’t enter any exercise protocol and did not receive any other anti-depressant therapy. HPLC-fluorometric detection assay was used to measure 24-hour urine MHPG sulfate values. The data was analyzed with t-test and Pierson’s correlation tests.

**Results:** Twenty hour urine MHPG sulfate increased from 1.93 (±0.59) to 4.66 (±0.85) micromole in case group (P ≤ 0.001), and in control group from 1.67 (±0.58) to 1.80 (±0.58) micromole. Increase of 24-hour urine MHPG sulfate and increasing of maximum oxygen consumption showed significant positive correlation (r = 0.65), and a significant negative correlation (r = 0.65) was observed between urine MHPG sulfate and Hamilton Rating Scale for Depression (Ham-D) score.

**Conclusion:** Aerobics training in exhilarating environments shows desirable influence over reduction of depression. This reduction of depression is correlated with MHPG sulfate elevation.

## Introduction

The pattern of diseases in developing countries has changed from infectious to non-infectious diseases such as cardiovascular disorders, diabetes mellitus, hypertension, cancer and mental disorders including depression. Inactivity and inappropriate lifestyle are the main reasons for occurrence of such diseases. World Health Organization (WHO) has estimated that until 2020, depression will be not only a major threat for human health but also will be the second factor of mortality and disability (1). Approximately 15-40% of people suffering from major depression commit suicide and nearly 10% of them will die. According to the reports, 1000 people die as result of suicide daily worldwide. In addition, the rate of attempted suicide is 8-10 times higher than death rate and it is more common in women than in men (1). Many studies have shown that depression can decrease some neurotransmiters of the central nervous system (CNS) such as norepinephrine (2-4). The main metabolite of norepinephrine- 3-Methoxy- 4-Hydroxyphenylglycol sulfate (MHPG sulfate) is released in the presynaptic terminals (5,6), and transmitted from the blood-brain barrier and then is excerted as a sulfate component in the urine (6,7). This component can be an index in the diagnosis of depression (3,7).

Previous studies have reported that urine MHPG sulfate is a more sensitive index for diagnosis of depression rather than urine metanephrine and normetanephrine (3,8).

People who exercise daily will suffer less from depression (1,3,9,10), and some studies have reported increase of MHPG sulfate in the brain following physical activity (3,6,11,12). Williams et al. reported that running on treadmill can increase the level of MHPG sulfate because of activation of the CNS in rats (12). Beckmann et al. showed that urinary MHPG sulfate increases by physical activity in depressed patients (13).

According to the investigations about depression, it seems that most researchers have used walking and running in sporting field or running on treadmill as a training protocol (12,14,15). Most of these studies have been done on depressed patients who aged more than 40 years (4,9,15), whereas there is a trend for depression to be increasing in prevalence at an earlier age (16), and the largest portion of suicidal attempts in depressed patients has been reported to occur in patients with age range of 15-24 years (17). Therefore, with respect to situation of these patients who need an exhilarating milieu and the effect of working out in attractive environment like buoyant milieu, the first purpose of this study was to determine the effect of six-week intermittent walking in the water on the rate of 24-hour urine MHPG sulfate and the severity of clinical depression in depressed girls. The second purpose of this research was to answer this question that "Can we use urine MHPG sulfate as an indicator of treatment effect in depression?"

## Materials and Methods

One hundred fifty-two female high school students (age range, 15-18 years) were assessed by two psychiatrists to document the presence of major depressive disorder (MDD) according to DSM-IV-TR criteria. Those with other simultaneous psychiatric disorders such as anxiety disorders, psychotic disorders, substance abuse or dependency, personality disorders, and bipolar mood disorder in depressive phase were excluded. One psychiatrist performed Hamilton Rating Scale for Depression (Ham-D) to assess the severity of depression. The Ham-D is the most commonly used valid and reliable observer-rated depression rating scale (18). Twenty-four subjects with a score of ≥ 18 were included into the study. They were divided randomly into experimental group (12 cases) or control group (12 cases). In the next phase, Maximum Oxygen Uptake (VO2 max) was measured by Bruce test in order to evaluate the aerobics tolerance. The subjects' anthropometric variables included height, age, body mass, VO2 max, body fat, fat mass, lean body mass, and body mass index. In order to measure the urinary level of MHPG sulfate, 24-hour urine was collected.

For the subjects in case group, a pool walking exercise program was implemented. Subjects of control group did not participate in any exercise program and did not receive any anti-depressant treatment. The pool walking exercise was carried out in a pool with 15 meters width. The water height in the pool was considered as much as 70 to 80% of the cases' height, and they walked with respect of their height in determined water height. Pool walking exercise was implemented for 6 weeks and 3 sessions every week. The distance of pool walking was increased gradually with regard to over load principle, the training sets and training cycle (19). The experimental group were divided to some subgroups and in each subgroup, those who had similar VO2 max walked in the water with equal intensity and rhythm. The activity intensity was constantly about 60-70% of maximum heart rate during 6 weeks. After every set, the activity intensity was controlled with pulse rate. On the average, the cases walked every 30-meter distances with aforesaid intensity in 50 to 60 seconds. The relaxation time between every walking was 30 to 40 seconds. The break time between the sets was 5 to 6 minutes in order to allow heart rate to return to primary situation. Generally, the participants exercised two times daily during the first week, but for the next weeks they exercised three times daily. In addition, the number of repeating of pool walking in 30-meter lengths from the first week until the 6th week was 34, 75, 24, 92, 112 and 131, respectively. The subjects in experimental group walked a total of 14,850 meters during 18 sessions of pool walking.

At the end of six-week exercise program, Ham-D and Bruce testing were performed and other anthropometric variables like the ones in pretest process were measured again for both groups. Twenty four-hour urine samples were collected once more with a method similar to pretest measurement. We used chromatography fluorometric (HPLC-fluorometric detection) method to determine 24-hour urine MHPG sulfate.

*Statistical analyses*

Since Kolmogorov-Smirnov testing showed a normal distribution, parametric statistical tests were used to analyze data. Dependent t-test was used to determine the changes of Ham-D and 24-hour urine MHPG sulfate between pre-test and post-test in experimental group and independent t-test was used to determine the difference between the two groups. Pierson correlation test was used for assessment correlation between Hamilton scale and 24-hour urine MHPG sulfate in experimental and control groups. Significance level (p value) was set as 0.05.

## Results

Table 1 presents mean and standard deviation (±SD) of anthropometric variables. There were no significant differences between the groups at the beginning of the research regarding these variables. Table 2 shows the amount of 24-hour urine MHPG sulfate in experimental group. As shown, mean (±SD) level of 24-hour urine MHPG sulfate increased from 1.93 (±0.59) micromole in pre-test to 4.66 (±0.85) micromole in post-test state. In control group, this figure changed from 1.67 (±0.58) to 1.80 (±0.58) micromole. There was a statistically significant increase in urine MHPG sulfate in experimental group compared to control group (141.45% vs. 7.78%; P ≤ 0.001). In addition, in comparison with the control group the independent t-test showed a significant increase in 24-hour urine MHPG sulfate and VO2 max and significant decrease (P ≤ 0.001) regarding Ham-D score in experimental group after 6 weeks pool walking exercise. Table 3 shows that after 6 weeks of exercise there was a positive correlation between 24-hour urine MHPG sulfate and VO2 max. Also, there was a negative correlation between 24-hour urine MHPG sulfate and Ham-D score.

## Discussion

The most important finding of this research is the considerable effect of exercising in exhilarating milieu on improvement of indices related to depression: urine MHPG sulfate, Ham-D, and VO2 max in depressed female teenagers. Increase in cardiovascular performance which was resulted from exercising program showed direct correlation with changes of urine MHPG sulfate, and had a negative correlation with Ham-D score.

Depression is one of the most common mood disorders. Considering that there are millions of people who suffer from depression all over the world, many researchers made a lot of effort to find the etiology. We can mention that the most noticeable reason of considerable Depression is one of the most common mood disorder. Considering that there are millions of people who suffer from depression all over the world, many researchers made a lot of effort to find the etiology. We can mention that the most noticeable reason of considerable prevalence of depression that attracted many researchers' attention was the biological etiology and the role of neurotransmitters such as norepinephrine. Researchers believe that norepinephrine has important roles in human mental states such as temper, feeling of happiness, and vegetative functions such as sleeping, appetite and reactions to stress (4). In this case, 24-hour urine MHPG sulfate which is one of the main metabolites of norepinephrine has been paid more attention (2,3,5,6,11). Findings indicate that measurement of urine MHPG sulfate (which is the most sensitive catecholamine metabolite in relation with depression) can show the variation of norepinephrine and brain neural activity during mood changes (2,3,5). The findings of this research also showed that after 6 weeks intermitting walking in water, the rate of 24-hour urine MHPG sulfate in experimental group increased significantly. These findings are compatible with former reports which showed the effect of physical activity on the increase of urine MHPG sulfate (2,3,5,6,11). According to our study, there are a few researches similar to one that we embarked on it (the effect of aerobics activity on the rate of urine MHPG sulfate in depressed people) and this problem made it difficult to comment upon the exact results. Nevertheless, researchers believe that aerobics exercising make hormonal (increase of endorphin, serotonin and norepinephrine) and physiological changes (increased blood flow, decreased muscle contraction, and increased neurotransmitter efficiency); emotional stress will be reduced, happiness and spiritual joy and positive emotional states will be reinforced (20). Neurotransmitters such as norepinephrine will be decreased in depression and release of norepinephrine from sympathetic nervous terminal will be increased by exercise (7). That is why physical activity can reduce the severity of depression with changing the amount of norepinephrine.

**Table 1:** Mean and standard deviation of studied variables in experimental and control groups

Variable	Age	Weight (kg)	Height (cm)	Fat (percent)	Mass Body index (square kilogram)	Lean Body Mass (kilogram)	Fat mass (kilogram)	VO2max (Milliliter/ kilogram/minutes)
Group								
Experimental	16.91 ± 1.03	54.94 ± 9.38	161.14 ± 6.37	23.29 ± 4.44	21.18 ± 3.87	41.78 ± 5.74	13.98 ± 5.82	31.32 ± 5.45
Control	16.83 ± 0.82	49.94 ± 9.92	157.23 ± 4.01	21.35 ± 3.71	20.35 ± 3.66	38.96 ± 5.81	12.91 ± 7.64	31.26 ± 6.02

**Table 2:** Mean (standard deviation) of studied indices before and after pool walking in experimental and control groups

Group and Index	Stages	Before exercise Mean (±SD)	After 6-week exercise Mean (±SD)
MHPG Urine Sulfate (µmol/24 hours)	Experimental	1.93 (±0.59)	‡ † 4.66 (±0.85)
	Control	1.67 (±0.58)	1.80 (±0.58)
Ham-D score	Experimental	30.15 (±7.62)	‡ † 14.08 (±5.79)
	Control	29.58 (±7.25)	25.58 (±9.72)
VO2max (_ml.min_-1_.kg_-1)	Experimental	31.32 (±5.45)	‡ † 38.37 (±4.85)
	Control	31.26 (±6.02)	30.50 (±5.94)

†* Shows significant differences in comparison to pretest (P ≤ 0.001)*

‡ *shows significant differences in comparison to control group (P≤0.001)*

**Table 3:** Pearson correlation coefficient of 24-hour urine MHPG sulfate and Hamilton testing score after 6 weeks of pool walking exercise

Variables	Ham-D score	*P value*	VO2 max	*P*
24-hour urine MHPG sulfate	-0.52	0.042	0.65	0.010

On the other hand, we can attribute the increase of urine MHPG sulfate and reduction of depression to the aerobic exercises performed. The findings of this research indicate that there is a significant increase (22.51 percent) in VO2 max in experimental group in comparison with control group. This finding confirms the previous reports based on the relationship of aerobic performance with depression and related indices (13,19). Norepinephrine level increases with exercise intensity above 50% of VO2 max (7). On the other hand, this research is different from other researches on depression for type of exercise implemented (walking in the water). It is certainly obvious that depressed patients will response to exercising, but training in the exhilarating and floating environments of water can have exclusive influence. Many studies suggest that floating in water is an effective factor for improving mood. They also indicate that training in water can influence further benefit than the land, so can cause the reduction of injury, because in the water weigh will reduce, somehow that in 1.2 meter depth 50 percent of the pressure to the joint will be reduced (21). In addition, in this research it was clearly defined that after 6 weeks of walking in the water, there was a significant negative correlation between the increase of urine MHPG sulfate and reduction of Ham-D score, and a significant direct correlation between urine MHPG sulfate and VO2 max. Results of this research are in agreement with previous studies that showed relationship between increases of MHPG sulfate and improvement of Ham-D score are in the same direction (22). Therefore, it can be concluded that urine MHPG sulfate is an important index for diagnosing the intensity of depression.

Findings of this research show that six-week intermittent walking in water has made a significant decrease (53.07%) in Ham-D. Both the result of this research and other researches showed significant improvement of depression after aerobic activity (9,21). Probably, the main reason of this improvement can be related to intensity, duration, the number of sessions and other physiological variables. Slawson indicates that intensity of physical activity is the main factor for improving mild and moderate depression, and he believes that walking with the rate of 70 to 80 percent of maximum heart rate for 3 sessions in week can reduce depression as the same seen in pharmacotherapy (15). Scully has reported that physical activity more than 4 sessions in a week can increase anxiety, nervousness and depression (23). But if exercise is done for 2 or 3 sessions in a week, it can have a positive effect on mood states. Some researchers recommend that people who want to do exercises to cure their depression, should do aerobic exercises for 3 sessions in a week (2). Researchers report that short-term physical activity, for example exercising for 10 days, can improve depressed mood, but for curing the major depression aerobic exercises should be done in long-term periods and combined with pharmacotherapy (4,14).

In summary, based on the findings of this research, it can be concluded that aerobic exercises in exhilarating milieu has a desirable effect on related indices with depression (urine MHPG sulfate, Ham-D score, and VO2 max) in moderate depressed female teenagers. Furthermore, we can use urine MHPG sulfate as an indicator of treatment response in depression.

## Authors' Contributions

VDR conceived and designed the evaluation, performed the statistical analysis and drafted the manuscript. MP helped to conceived and designed the evaluation, performed Ham-D and parts of laboratory experiments, and interpreted the research results. ZM performed parts of the research methods and carried out the training protocol during 6 weeks. All authors read and approved the final manuscript.

## Notes

**Declaration of Interest:** None.

**Clinical Trial Registration: **URL: http://www.Irct.ir/Unique identifier: IRCT201104251457N8

the rate of MHPG sulfate and the severity of depression.. Iran J Psychiatry Behav Sci 2011; 5(2): 26-31.

## References

[1] World Health Organization (WHO) (2011). Depression: What is depression?. http://www.who.int/mental_health/management/depression/definition/en/.

[2] Doyne EJ, Ossip-Klein DJ, Bowman ED, Osborn KM, McDougall-Wilson IB, Neimeyer RA (1987). Running versus weight lifting in the treatment of depression. J Consult Clin Psychol.

[3] Pilu A, Sorba M, Hardoy MC, Floris AL, Mannu F, Seruis ML (2007). Efficiency of physical activity in the adjunctive treatment of major depressive disorders. J Clin Pract Epidemiol Ment Health.

[4] Dimeo F (2001). Aerobic exercise lifts depression in treatment resistant patients. J Br JSP Med.

[5] Elsworth JD, Roth RH, Redmond DE Jr (1983). Relative importance of 3-methoxy-4-hydroxyphenylglycol and 3, 4-dihydroxyphenylglycol as norepinephrine metabolites in rat, monkey, and humans. J Neurochem.

[6] Peyrin L.J (1990). Urinary MHPG sulfate as a marker of central norepinephrine metabolism: a commentary. Neural Transm Gen Sect.

[7] Peyrin L, Pequignot JM, Lacour JR, Fourcad J (1987). Relationships between catecholamine or 3-methoxy 4-hydroxy phenylglycol changes and the mental performance under submaximal exercise in man. Psychopharmacology.

[8] Salah Eidin Zaki M, Elbatrawy AN (2009). Catecholamine Level and Its Relation to Anxiety and Depression in Patients with Vitiligo. J Egypt Women Dermatol Soc.

[9] Salmon P (2000). Effects of physical exercise on anxiety, depression, and sensitivity to stress: a unifying theory. Clin Psychol. Rev.

[10] Herman S, Blumenthal JA, Babyak M, Khatri P, Craighead WE, Krishnan KR, Doraiswamy PM (2002). Health Psychol.

[11] Swamp R.A, Norvell N, Graves JE, Pollock. ML (2000). High versus Moderate Intensity Aerobic Exercise in Older Adults: Psychological and Physiological Effects. J Aging Phys Activ.

[12] Dunn AL, Reigle TG, Youngstedt SD, Armstrong RB, Dishman RK (1996). Brain norepinephrine and metabilites after treadmill and wheel running in rats. Med Sci Sports Exerc.

[13] Beckmann H, Ebert MH, Post R, Goodwin FK (1979). Effect of moderate exercise on urinary MHPG in depressed patients. Pharmakopsychiatr Neuropsychopharmakol.

[14] Knuben K, Reischies FM, Adli M, Schlattmann P, Bauer M, Dimeo F (2007). Randomized controlled study on the effects of a short-term endurance training program in patients with major depression. Commentary. Br J Sports med.

[15] Slawson D (2005). Aerobic exercise effective for mild to moderate depression. Am Fam Physician.

[16] Joyce PR, Oakley-BrowneMA, WellsJE, BushnellJA, Hornblow AR (1990). Birth cohort trends in major depression: increasing rates and earlier onset in New Zealand. Journal of Affective Disorders.

[17] Blumenthal SJ, Kupfer DJ (1988). Overview of early detection and treatment strategies for suicidal behavior in young people. J Youth Adolesc.

[18] Hamilton M, American Psychiatric Association (2000). Hamilton Rating Scale for Depression (Ham-D). Handbook of Psychiatric Measures.

[19] Dabidi Roshan V, Pourasghar M, Mohammadian Z (2009). Research Sport science journal.

[20] Peyrin L, Pequignot JM (1983). Free and conjugated 3-methoxy-4-hydroxyphenylglycol in human urine: Peripheral origin of glucuronide. J Psychopharmacology.

[21] Broman G, Quintana M, Lindberg T, Jansson E, Kaijser L (2006). High intensity deep water training can improve aerobic power in elderly women. Eur J Appl physiol.

[22] Shinkai K, Yoshimura R, Ueda N, Okamoto K, Nakamura J (2004). Associations between baseline Plasma MHPG levels and clinical responses with respect to milnacipran versus paroxetine treatment. J Clin sychopharmacol.

[23] Scully J, Kremer J, Meade MM, Graham R, Dudgeon K (1998). Physical exercise and psychological well being: a critical review. Br J Sports Med.

